# Endothelium-Derived 5-Methoxytryptophan Protects Endothelial Barrier Function by Blocking p38 MAPK Activation

**DOI:** 10.1371/journal.pone.0152166

**Published:** 2016-03-22

**Authors:** Ling-Yun Chu, Yi-Fu Wang, Huei-Hsuan Cheng, Cheng-Chin Kuo, Kenneth K. Wu

**Affiliations:** 1 Metabolomic Medicine Research Center, China Medical University Hospital, Taichung, Taiwan; 2 Institute of Cellular and System Medicine, National Health Research Institutes, Zhunan, Taiwan; 3 Institute of Molecular Medicine, National Tsing Hua University, Hsinchu, Taiwan; 4 Graduate Institute of Clinical Medical Science, China Medical University, Taichung, Taiwan; 5 Graduate Institute of Basic Medical Science, China Medical University, Taichung, Taiwan; 6 Department of Medical Sciences and Institute of Biotechnology National TsingHua University, Hsinchu, Taiwan; University of Kentucky, UNITED STATES

## Abstract

The endothelial junction is tightly controlled to restrict the passage of blood cells and solutes. Disruption of endothelial barrier function by bacterial endotoxins, cytokines or growth factors results in inflammation and vascular damage leading to vascular diseases. We have identified 5-methoxytryptophan (5-MTP) as an anti-inflammatory factor by metabolomic analysis of conditioned medium of human fibroblasts. Here we postulated that endothelial cells release 5-MTP to protect the barrier function. Conditioned medium of human umbilical vein endothelial cells (HUVECs) prevented endothelial hyperpermeability and VE-cadherin downregulation induced by VEGF, LPS and cytokines. We analyzed the metabolomic profile of HUVEC conditioned medium and detected 5-MTP but not melatonin, serotonin or their catabolites, which was confirmed by enzyme-linked immunosorbent assay. Addition of synthetic pure 5-MTP preserved VE-cadherin and maintained barrier function despite challenge with pro-inflammatory mediators. Tryptophan hydroxylase-1, an enzyme required for 5-MTP biosynthesis, was downregulated in HUVECs by pro-inflammatory mediators and it was accompanied by reduction of 5-MTP. 5-MTP protected VE-cadherin and prevented endothelial hyperpermeability by blocking p38 MAPK activation. A chemical inhibitor of p38 MAPK, SB202190, exhibited a similar protective effect as 5-MTP. To determine whether 5-MTP prevents vascular hyperpermeability in vivo, we evaluated the effect of 5-MTP administration on LPS-induced murine microvascular permeability with Evans blue. 5-MTP significantly prevented Evans blue dye leakage. Our findings indicate that 5-MTP is a new class of endothelium-derived molecules which protects endothelial barrier function by blocking p38 MAPK.

## Introduction

Vascular endothelium is composed of a single layer of endothelial cells (ECs) lining the inner wall of the entire vasculature. ECs are active producers of vasoprotective molecules to defend against thrombosis, vasoconstriction and endothelial apoptosis. They are situated in a strategically important location, where they are in close contact with circulating blood, its constituents and various invading agents. They possess tight intercellular junctions that act as a barrier to restrict the passage of blood cells, solutes and other substances into the vascular wall and the underlying tissues [1]. The barrier function is maintained by a number of junction protein complexes among which adherens junction is the major determinant [2]. The adherens junction complex comprises vascular endothelial (VE)-cadherin, catenins and plakoglobin. VE-cadherin is the anchor protein with a typical extracellular cadherin domain that mediates tight junction formation via homophilic interactions [3]. The cytoplasmic domain of VE-cadherin binds p120, β-catenin and plakoglobin, which in turn bind α-catenin. α-Catenin interacts with the actin cytoskeleton, linking the adherens junction complex with the cytoskeleton system [2,3]. The disruption of endothelial barrier function is required for inflammatory responses. Endothelial barrier function is disrupted by diverse pro-inflammatory factors, notably vascular endothelial growth factor (VEGF), tumor necrosis factor-α (TNF-α), interleukin-1β (IL-1β), and lipopolysaccharide (LPS), resulting in increased endothelial permeability [2,4]. VEGF was reported to disrupt the adherens junction by inducing VE-cadherin internalization and degradation [5] or causing dissociation of the intracellular domain of VE-cadherin from catenins through tyrosine phosphorylation [6,7]. TNF-α and LPS were reported to disrupt the barrier function by reducing surface expression of VE-cadherin [8,9].

Disruption of barrier function by pro-inflammatory mediators results in vascular inflammation and damage leading to vascular diseases such as atherosclerosis, vascular remodeling and restenosis [10,11]. Protection of endothelial barrier function and defense against inflammation are critical in maintaining normal vascular integrity and prevention of chronic vascular diseases. We previously reported the detection of soluble factors (“cytoguardin”) which inhibit cyclooxygenase-2 (COX-2) expression by blocking NF-κB and p300 HAT activation induced by pro-inflammatory mediators [12,13]. Chemical identity of cytoguardin was resolved by comparative metabolomics. It is a metabolite of L-tryptophan, i.e. 5-methoxytryptophan (5-MTP) which is synthesized via a novel metabolic pathway [14]. Two enzymatic steps for its biosynthesis in fibroblasts have been identified: 1. tryptophan hydroxylase (TPH) which catalyzes conversion of L-tryptophan to 5-hydroxytryptophan (5-HTP); and 2. hydroxyindole O-methoxytransferase which converts 5-HTP to 5-MTP [14]. This pathway shares with melatonin biosynthesis at several enzymatic steps but has a major difference: melatonin biosynthesis requires decarboxylation of 5-HTP whereas cytoguardin (5-MTP) synthesis does not. This results in a distinct structural feature of 5-MTP in that it retains the propionic acid side chain, while melatonin and its major catabolite, 5-methoxytryptamine do not. 5-methoxy and 8-propionic acid side chains of 5-MTP were considered to be crucial for its potent actions against COX-2 expression and NF-κB activation when compared to melatonin and its catabolites [15,16]. Since vascular endothelium faces inflammatory challenges from LPS, cytokines and growth factors in the circulating blood and serves as the first line of defense, we postulated that ECs produce 5-MTP to protect endothelial barrier function and defend against vascular inflammation.

## Materials and Methods

### Materials

LPS was purchased from Sigma-Aldrich. Recombinant human VEGF, IL-1β and TNF-α were purchased from Cell Signaling Technology. Rabbit monoclonal antibodies against VE-cadherin, phospho-p38 (Thr180/Tyr182), p38, phospho-Src, Src, phospho-ERK1/2, ERK1/2, ICAM-1 and VCAM-1 were purchased from Cell Signaling Technology. Inhibitor of p38, SB202190, was purchased from Cell Signaling Technology. Rabbit polyclonal antibodies against 5-MTP was purchased from Abcam. Horseradish peroxidase-conjugated anti-mouse and anti-rabbit IgG were purchased from Santa Cruz.

### Preparation of 5-MTP

Two different sources of 5-MTP have been used in this study. DL-5-MTP, a mixture of L-form and D-form 5-MTP, was purchased from Sigma-Aldrich, and L-5-MTP was custom-synthesized by AstaTech (AstaTech Inc, Bristol, PA). Purity of L-5-MTP and DL-5-MTP was verified by LC-MS. For in vitro experiments, a DL-5-MTP stock solution (50 mmol/L) was prepared in dimethylsulfoxide and stored at -20°C. For in vivo permeability assay, L-5-MTP was freshly dissolved in saline (0.5 ml, pH adjusted to 7.4).

### Cells

Primary HUVECs were purchased from Bioresource Collection and Research Center (Hsinchu, Taiwan) and maintained in HUVEC growth medium (EBM-2, Lonza) with full supplements (2% fetal bovine serum, 0.4% human fibroblast growth factor-2, 0.1% vascular endothelial growth factor, 0.1% R^3^-insulin-like growth factor-1, 0.1% human epidermal growth factor, 0.04% hydrocortisone, 0.1% ascorbic acid, 0.1% GA-1000). Only passages 3 to 6 cells were used. Cells were treated with DL-5-MTP, conditioned medium or chemical inhibitors for 30 minutes before addition of inflammatory cytokines. At the indicated time point, cells were lysed with ice-cold lysis buffer (20 mmol/L Tris-HCl, pH 7.5, 150 mmol/L NaCl, 1 mmol/L EDTA, 1 mmol/L EGTA, 1% CHAPS, 2.5 mmol/L sodium pyrophosphate, 1 mmol/L β-glycerophosphate, 1 mmol/L Na3VO_4_) and protease inhibitors (1 tablet in 10mL lysis buffer, Roche) immediately.

### Preparation of HUVEC conditioned medium (CM)

HUVECs were grown to 80% confluence and the medium was removed and replaced with fresh medium. Cells were incubated for another 24 hours and the CM was collected. The collected CM was centrifuged at 1,000g for 5 minutes; the supernatant was collected and stored at 4°C for no more than 1 week. For rescuing experiments, cells in 6-well plates were pretreated with 1ml CM for 30 minutes before addition of inflammatory cytokines. For measurement of 5-MTP in CM, HUVECs were seeded in 6-well plates to 80% confluence and the medium was replaced with 1ml fresh medium and incubated with or without pro-inflammatory cytokines for 24 hours. CM was then collected and stored at -20°C.

### Immunoblotting

Cells were scraped and lysed in lysis buffer on ice for 5 minutes. Lysates were then centrifuged at 12,000g for 5 minutes to remove cell debris. Supernatants containing 25μg of protein were mixed with Laemmli sample buffer (Bio-Rad) and boiled for 5min before being subjected to electrophoresis in 4–15% sodium dodecyl sulfate-polyacrylamide gels and then transferred to polyvinylidene difluoride membranes (Millipore). For detection, membranes were blocked with 5% bovine serum albumin in 25 mmol/L Tris-HCl, pH 7.4, 150 mmol/L NaCl, and 0.05% Tween 20 for 1 hour at 22°C and then incubated with 1:1000 diluted primary antibodies overnight at 4°C. Membranes were incubated with 1:3000 diluted horseradish peroxidase-linked secondary antibodies for 1 hour at 22°C and then developed with enhanced chemiluminescence substrate (Thermo Scientific). The blot was exposed with a luminescent image analyzer (ImageQuant LAS 4000, GE Healthcare) and quantified by ImageJ software (National Institute of Health).

### Analysis of 5-MTP and related metabolites by liquid chromatography-mass spectrometry

The metabolites were analyzed using an ultraperformance liquid chromatography (Acquity UPLC system, Waters Corporation) coupled with a Xevo-ToF mass spectrometer (Waters) according to a method previously described.^14^ CM passed through a 3kD membrane, the filtrate was collected and applied to UPLC-MS.

### In vitro endothelial permeability assay

The in vitro permeability assay was performed with the In Vitro Vascular Permeability Assay Kit (Millipore). In brief, HUVECs at 100% confluence were harvested and resuspended with growth medium. Cells were seeded into the insert plate (1 x 10^5^ cells/ well) and then incubated for 72 hours to form a monolayer. The HUVEC monolayers were then treated with inflammatory cytokines at indicated concentrations for 24 hours. After treatment, FITC-Dextran (~70kDa) solution was added onto the monolayers for 20 minutes. Medium containing FITC-Dextran that crossed the monolayer in the receiver tray was analyzed by a fluorescence plate reader (Synergy H1 Hybrid Reader, BioTek) with 485 nm excitation and 535 nm emission filters.

### Pulmonary microvascular permeability assays in mice

The Institutional Animal Care and Use Committee of National Health Research Institutes (NHRI), Taiwan approved all experimental procedures. The experimental animal center at NHRI was accredited by the Association for Assessment and Accreditation of Laboratory Animal Care International (AAALAC). The basic animal maintenance conditions including lighting, light cycles, feeding and housing cleaning are in keeping with the rules of AAALAC. To determine microvascular leakage, we used Evans blue assay and analysis of cell counts and protein levels in bronchoalveolar lavage fluid (BALF). Approximately 12-week-old male C57BL/6 wild-type (National Laboratory Animal Center, Taiwan) mice were used. Evans blue dye (20mg/kg) was injected into the tail vein of mice which had been treated with or without LPS (60mg/kg) for 24 hours in the presence or absence of L-5-MTP (23.4mg/kg). Lungs were perfused, removed and Evans blue was extracted with 1ml of formamide overnight at 55°C. Evans blue was measured by spectrometry at 620 nm. To analyze cell and proteins in BALF, mice were sacrificed at 24 hours and tracheotomy was performed. The lung tissues were lavaged with 1mL PBS for 3 times and the BALF was collected. The recovered BALF was filtered through a layer of sterile gauze, centrifuged (1000 g, 4°C, 5 minutes), and resuspended. Total cell number in BALF was counted using a standard hemocytometer. Protein concentration of BALF was measured by using the method of Bradford. Albumin concentration in BALF was determined using a mouse albumin ELISA kit (Bethyl Laboratories).

### Measurement of 5-MTP with enzyme-immunoassay

A mixture of DL-5-MTP (Sigma) standards or samples and DL-5-MTP conjugated with HRP were added to 96-well microtiter plates precoated with 5-MTP antibodies and incubated at 4°C overnight. After washing, tetramethylbenzidine was added and incubated at room temperature for 30 minutes. Reaction was stopped with 0.1 mmol/L H_2_SO_4_ and the product was analyzed at 450 nm. The calibration curve which was constructed for each experiment was established by using pure DL-5-MTP at concentrations from 0.1~50 μmol/L.

### MTT Assay

HUVECs were seeded on 96-well plates (1 x 10^5^ cells/ well), incubated for 24 hours and treated with inflammatory cytokines at indicated concentration for another 24 hours. After treatment, cells were incubated with 100μL fresh growth medium plus 10μL 12 mol/L MTT (3-(4,5-dimethylthiazol-2-yl)-2,5-diphenyltetrazoliumbromide) stock solution for 4 hours. After incubation, remove 85μL medium and then add 50μL dimethylsulfoxide for 10 minutes at 37°C. The amount of insoluble formazan converted from MTT by cells was then determined by the absorbance at 540 nm with a plate reader (Synergy H1 Hybrid Reader, BioTek).

### Reverse transcription-polymerase chain reaction (RT-PCR)

HUVECs were seeded on 6-well plates (1 x 10^5^ cells/ well) and incubated for 48 hours. Cells were then treated with inflammatory cytokines at indicated concentration for another 24 hours. To isolate RNA, cells were lysed with 1mL Trizol/ well for 5 minutes. RNA was purified from lysates with Direct-zol RNA MiniPrep kit (Zymo research). First-strand cDNA was synthesized by incubating 1μg RNA and SuperScript III First-Strand Synthesis SuperMix (Invitrogen) for 30 minutes at 50°C. TPH-1 and TPH-2 DNA were amplified by mixing 1μL cDNA, 1μL 10 μmol/L primer each and 47μL HotStar PCR SuperMix (GeneDirex) for 35 cycles (94°C for 20 seconds, 57°C for 20 seconds, 72°C for 45 seconds) with a MultiGene Thermal Cycler system (Labnet International). The primer sequences for TPH-1 are: forward CGT CCT GTG GCT GGT TAC TTA and reverse AGT AGC ACG TTG CCA GTT TTT G. The primer sequences for TPH-2 are: forward TTG GAG AAT TAA AGC ACG CCC and reverse ACA ATG AGT GGT TAT CTG CCA T.

### Statistical Analysis

Values were expressed as mean ± SD. Differences between groups were analyzed using One Way ANOVA with SigmaStat software (Systat Software, Inc.). *P* < .05 was considered statistically significant.

## Results

### 5-MTP Protects HUVEC VE-cadherin and Barrier Function

**Fig 1:**
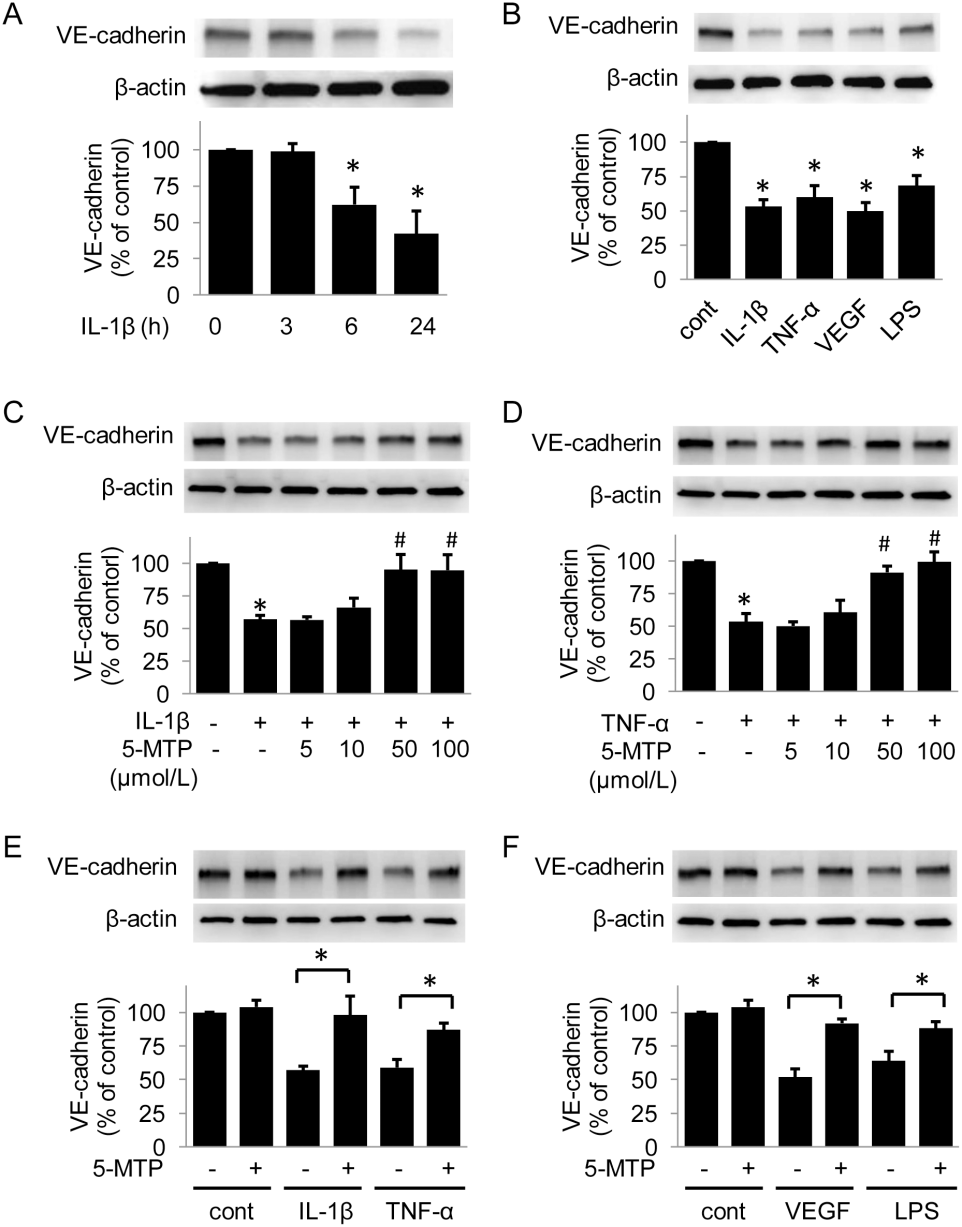
5-MTP protects VE-cadherin. (A) IL-1β Suppressed VE-cadherin Expression in a Time-dependent Manner. (B) VE-cadherin expression was suppressed by VEGF and pro-inflammatory cytokines at 24 hours. (C) and (D) 5-MTP inhibited IL-1β- (C) or TNF-α-induced (D) downregulation of VE-cadherin in a concentration-dependent manner. (E) and (F) 5-MTP protected VE-cadherin from VEGF and pro-inflammatory cytokines. Upper panels show representative Western blots and lower panels the densitometry. * indicates *P* < .05 compared to control and # indicates *P* < .05 compared to IL-1β or TNF-α group.

To determine whether inflammatory cytokines reduce total VE-cadherin expression, we first analyzed VE-cadherin expressions in HUVECs treated with IL-1β. IL-1β suppressed VE-cadherin expression in a time dependent manner up to 24 hours (Fig 1A). Furthermore, the extent of VE-cadherin suppression by VEGF, TNF-α and LPS was similar to that by IL-1β at 24 hours (Fig 1B). Since we have identified 5-MTP as an anti-inflammatory factor, we evaluated the effect of 5-MTP on cytokines-induced suppression of VE-cadherin. As shown in Fig 1C, pretreatment with 5-MTP for 30 minutes prevented IL-1β-induced VE-cadherin downregulation in a concentration-dependent manner with a significant effect at 50 and 100 μmol/L. 5-MTP inhibited TNF-α-induced VE-cadherin reduction in a similar concentration-dependent manner (Fig 1D). 5-MTP at 100 μmol/L also significantly prevented VEGF- and LPS-induced VE-cadherin suppression to an extent comparable to cytokine-induced suppression (Fig 1E and 1F).

**Fig 2:**
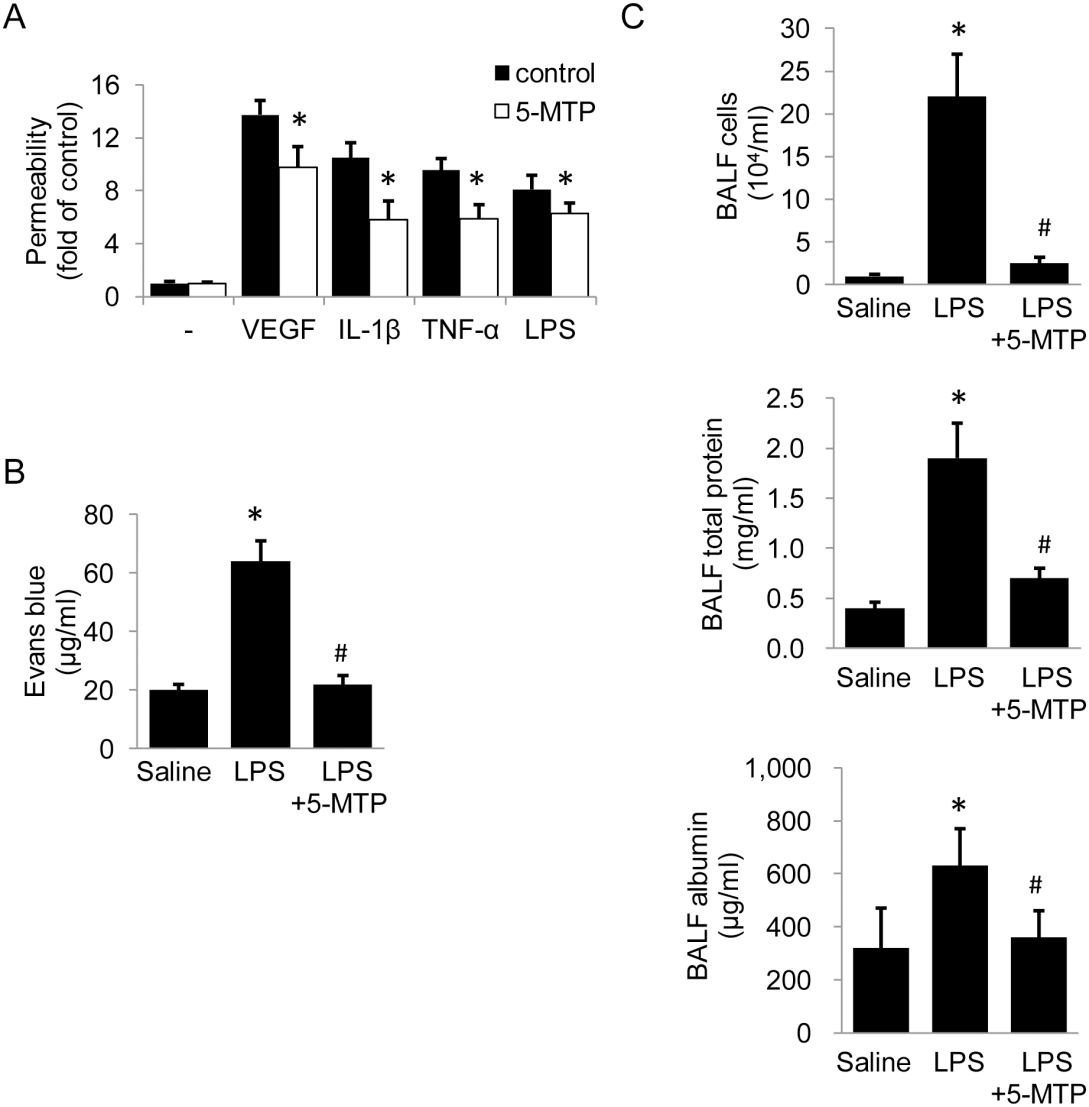
5-MTP protects barrier function in vitro and in vivo. (A) Analysis of 5-MTP (100 μmol/L) effect on endothelial permeability by an in vitro permeability assay with FITC-labeled dextran. Each bar denotes mean ±SD of three independent experiments. * indicates *P* < .05. (B) Mice were injected with L-5-MTP (23.4 mg/kg) for 30 minutes prior to challenge with LPS (60 mg/kg) for 24 hours. Evans blue dye was used to analyze pulmonary microvascular permeability as described in Methods. The error bars denote mean ± SD (n = 8 mice/group). * indicates *P* < .05 compared to control and # indicates *P* < .05 compared to LPS group. (C) Mice were injected with L-5-MTP (23.4 mg/kg) for 30 minutes prior to challenge with LPS (60 mg/kg) for 24 hours. BALF was collected by lavage. Total number of leukocytes was determined by cell counting. Total protein and albumin were analyzed. The error bars denote mean ± SD (n = 5 mice/group). * indicates *P* < .05 compared to control and # indicates *P* < .05 compared to LPS group.

As VE-cadherin degradation is accompanied by increased endothelial permeability, we wondered if 5-MTP protects endothelial barrier function through preserving VE-cadherin. We performed in vitro endothelial permeability experiments and the results showed that VEGF, cytokines or LPS disrupted barrier function and increased endothelial permeability while pretreatment with 5-MTP significantly reduced permeability induced by each of the factors (Fig 2A). We next examined the effect of 5-MTP on permeability in vivo with a murine pulmonary microvascular permeability assay. LPS treatment increased vascular permeability which was blocked by 5-MTP administration (Fig 2B). To further test the effect of 5-MTP on permeability in vivo, we performed bronchoalveolar lavage in mice and measured total proteins, albumin, and cell counts in BALF. 5-MTP significantly reduced BALF total proteins, albumin and cell counts in mice treated with LPS (Fig 2C). These results indicate that 5-MTP protects barrier function by preserving VE-cadherin.

### Endothelial Cells Release 5-MTP into Conditioned Medium

**Fig 3:**
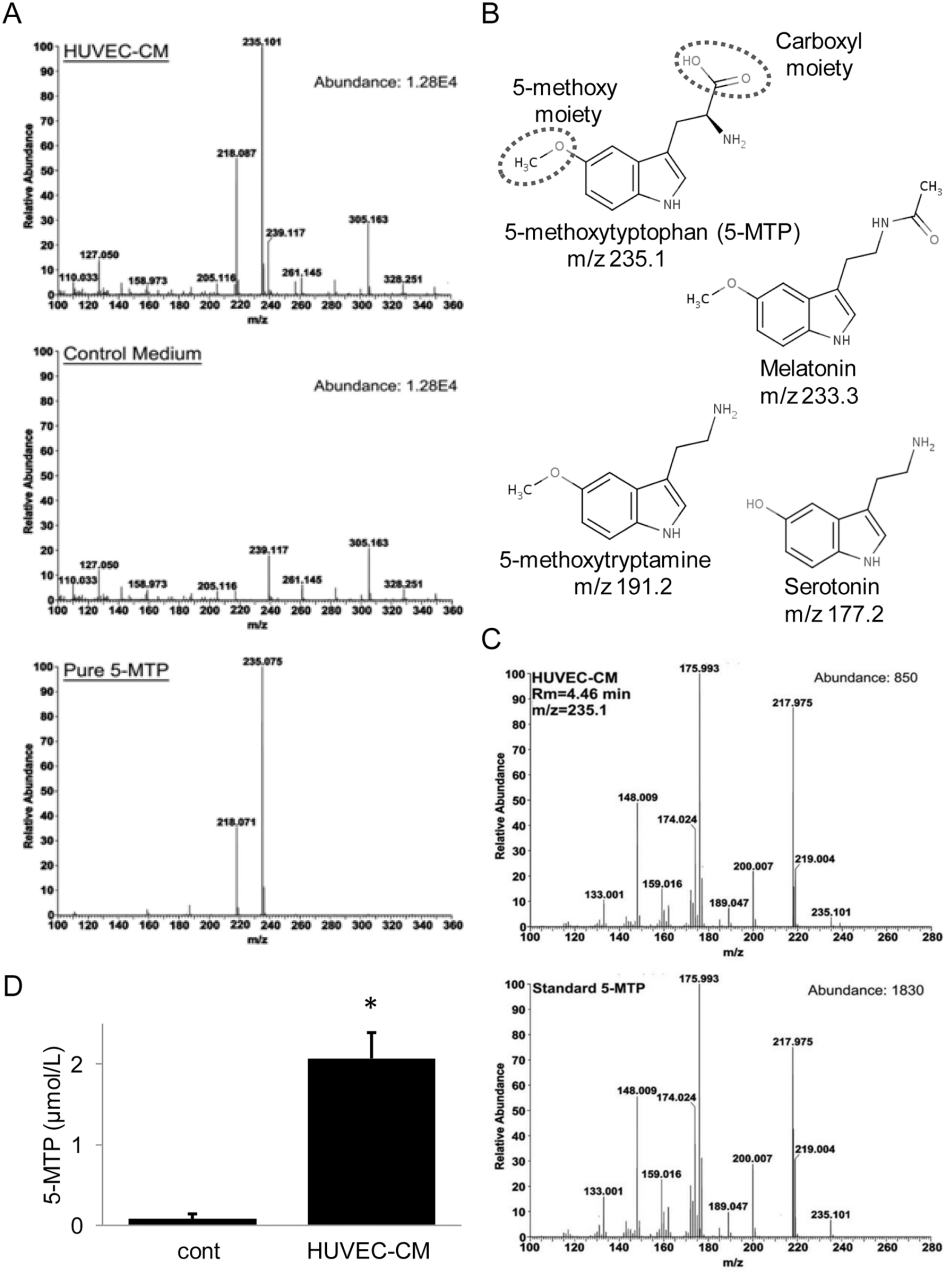
Endothelial cells release 5-MTP. (A) Comparison of single mass spectra of HUVEC-CM vs. control medium and pure 5-MTP by UPLC-QTof MS. (B) Comparison of the chemical structure of 5-MTP with that of major 5-HTP metabolites. The dashed circles depict the active groups of 5-MTP. Melatonin (N-acetyl 5-methoxytryptamine) possesses 5-methoxy but not the carboxyl group due to decarboxylation. It has weak anti-COX2 action. 5-methoxytryptamine, a major catabolic product of melatonin, is inactive. Serotonin (5-hydroxytryptamine) is also inactive. (C) Analysis of daughters of the m/z 235.1 peak in HUVEC-CM vs. that of pure 5-MTP by LC-MS-MS. (D) Measurement of 5-MTP in HUVEC-CM by ELISA. “cont” denotes control medium. * indicates *P* < .05.

To determine whether endothelial cells produce 5-MTP, we analyzed 5-MTP and related tryptophan metabolites in the conditioned medium (CM) of HUVECs by UPLC-QTof mass spectrometry. HUVEC-CM was passed through a 3 kDa filter and the filtrate was collected for metabolomic analysis using synthetic pure 5-MTP as a standard. The mass spectra of HUVEC-CM exhibited a prominent m/z 235.1 peak matching the m/z 235.1 peak of pure 5-MTP while the spectra of control medium did not have detectable m/z 235.1 (Fig 3A). There are no detectable m/z peaks which match other tryptophan metabolites such as serotonin (m/z 177.2), melatonin (m/z 233.3) or 5-methoxytryptamine (m/z 191.2), a key catabolite of melatonin (Fig 3B). To confirm that m/z 235.1 peak is 5-MTP, we analyzed its daughter profile by LC-MS-MS and compared with that of pure 5-MTP. The daughter profile of m/z 235.1 peak of HUVEC-CM was identical to that of pure 5-MTP (Fig 3C). To confirm 5-MTP in HUVEC-CM, we measured 5-MTP by ELISA. As shown in Fig 3D, HUVEC-CM contains > 20 folds 5-MTP over the control medium (2.07 ± 0.32 vs. 0.08 ± 0.06 μmol/L). These results indicate that HUVECs release a considerable amount of 5-MTP into CM without concurrent release of melatonin, serotonin or their catabolites.

### HUVEC-CM Prevents VE-cadherin Reduction and Endothelial Hyperpermeability Induced by Pro-inflammatory Mediators

**Fig 4:**
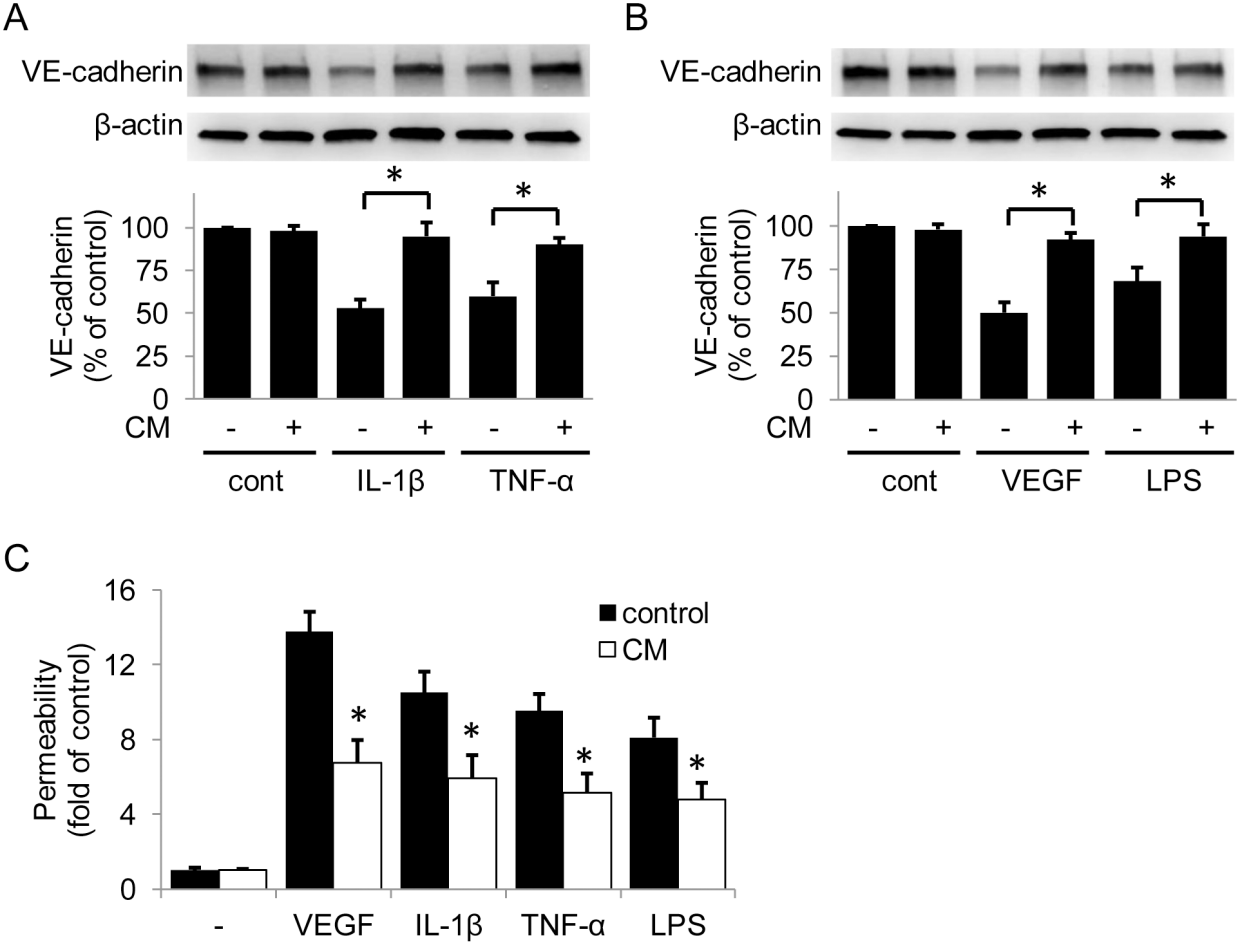
HUVEC-CM prevents VE-cadherin reduction and endothelial hyperpermeability induced by pro-inflammatory mediators. (A) and (B) HUVEC-CM protected VE-cadherin from VEGF and pro-inflammatory cytokines. The upper panel shows a representative Western blot and the lower panel the densitometry. * indicates *P* < .05. (C) Analysis of HUVEC-CM effect on endothelial permeability by an in vitro permeability assay with FITC-labeled dextran. Each bar denotes mean ±SD of three independent experiments. * indicates *P* < .05.

In view of the protective effect of 5-MTP on barrier function and the presence of 5-MTP in HUVEC-CM, we determined whether HUVEC-CM protects barrier function despite the presence of IL-1β, TNF-α, LPS or VEGF (Fig 4A and 4B). Corresponding to VE-cadherin preservation, incubation with HUVEC-CM prevented endothelial hyperpermeability induced by each of the pro-inflammatory mediators (Fig 4C). It is surprising that HUVEC-CM which contains ~2 μmol/L 5-MTP protects VE-cadherin as effectively as 50 μmol/L of synthetic 5-MTP. The reasons for this discrepancy are unclear but may be attributed to the presence of additional soluble factors in HUVEC-CM which are capable of protecting barrier function. Alternatively, 5-MTP in the HUVEC-CM may be in a conjugated form which is more active than synthetic 5-MTP.

### 5-MTP Protects Endothelial Barrier Function and VE-cadherin by Blocking p38 MAPK Activation

**Fig 5:**
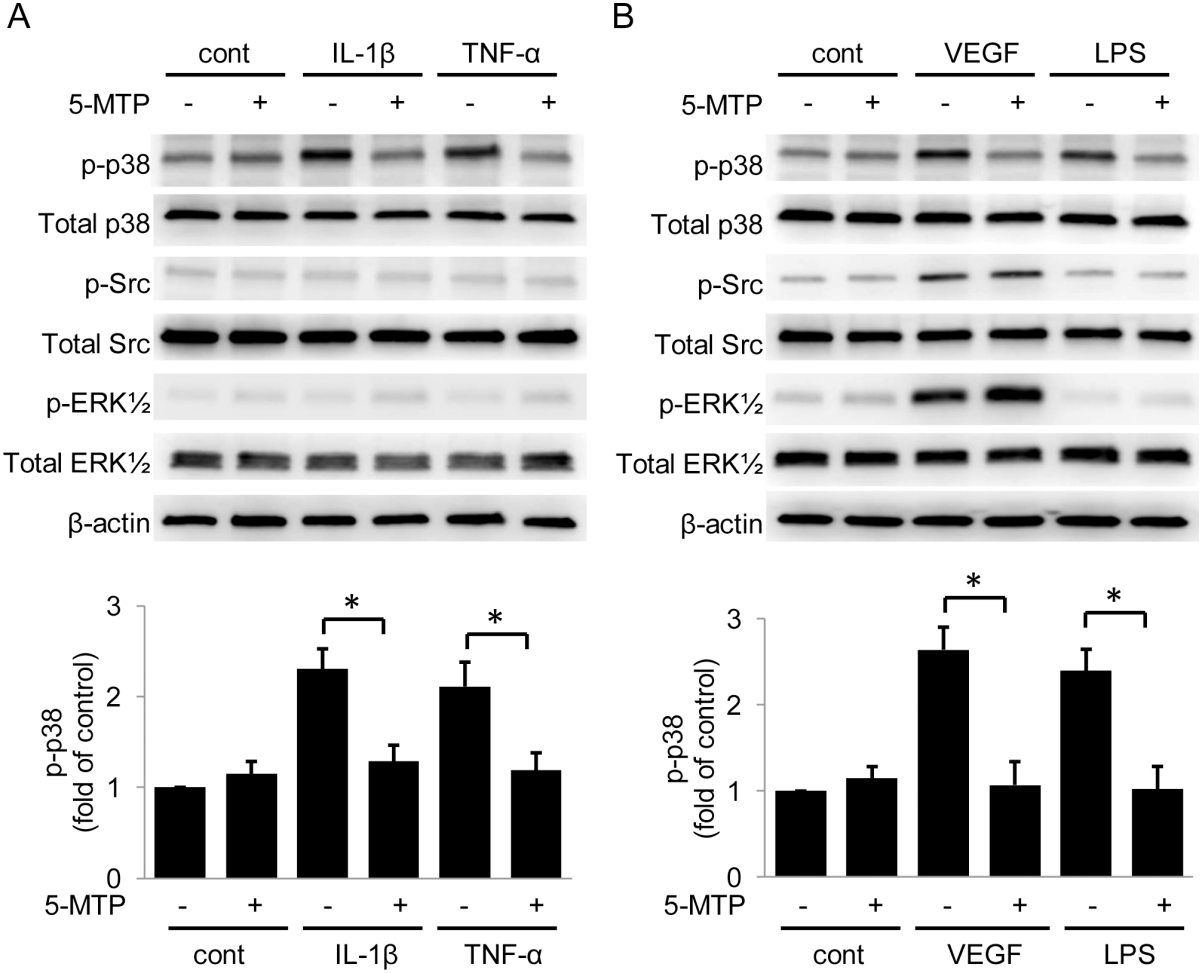
5-MTP blocks p38 MAPK activation. (A) and (B) Activation of p38, Src and ERK½ was determined in HUVECs treated with VEGF or pro-inflammatory cytokines. Upper panels show representative blots and lower panels show the densitometry of p-p38 MAPK. Error bars denote mean ± SD (n = 3). * indicates *P* < .05.

**Fig 6:**
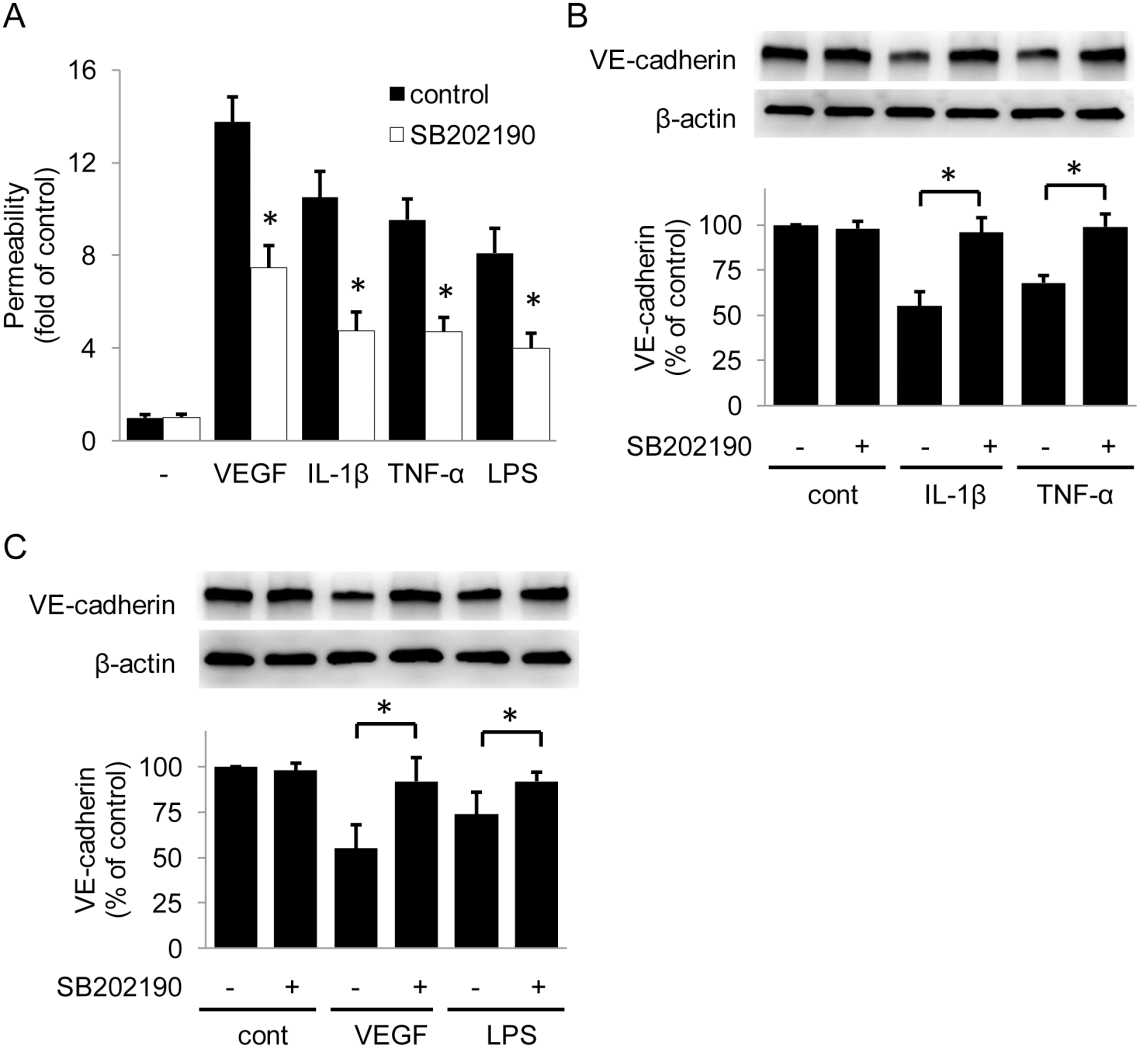
SB202190 protects HUVEC barrier function. (A) Pretreatment with SB202190 (10 μmol/L), a p38 inhibitor, attenuated VEGF- or pro-inflammatory cytokines-induced HUVECs permeability. Error bars denote mean ± SD (n = 3). * indicates *P* < .05 compared to control. (B) and (C) SB202190 protected VE-cadherin from VEGF and pro-inflammatory cytokines. Upper panels show representative blots and the lower panel, densitometry. Error bars denote mean ± SD (n = 3). * indicates *P* < .05.

Although it is well recognized that VEGF disrupts endothelial barrier primarily via Src family kinases (SFK), recent reports indicate that TNF-α, reactive oxygen species, infectious agents and environmental toxins increase endothelial permeability via p38 MAPK signaling pathway [17–21]. We thus examined whether 5-MTP regulates kinase activation. Consistent with previous reports, IL-1β, TNF-α, VEGF and LPS induced phosphorylation of p38 MAPKs (Fig 5A and 5B). 5-MTP at 100 μmol/L significantly inhibited p38 MAPK activation induced by IL-1β, TNF-α as well as VEGF and LPS (Fig 5A and 5B). None of the factors except VEGF activate Src or ERK½ kinase and 5-MTP had no inhibitory effect on VEGF-induced Src or ERK½ activation (Fig 5B). To confirm that the activation of p38 MAPK is required for mediating endothelial barrier disruption, we evaluated the effect of a pharmacological inhibitor of p38 MAPK, SB202190. SB202190 blocked endothelial hyperpermeability induced by IL-1β, TNF-α, LPS as well as VEGF (Fig 6A). SB202190 pretreatment also preserved VE-cadherin levels in HUVECs treated with pro-inflammatory mediators (Fig 6B and 6C). These results suggest that activation of p38 is required for downregulation of VE-cadherin and 5-MTP protects endothelial barrier function by preserving VE-cadherin via blocking p38 MAPK activation.

### 5-MTP Suppresses Pro-inflammatory Cytokines-induced ICAM-1/VCAM-1 expression in HUVECs

**Fig 7:**
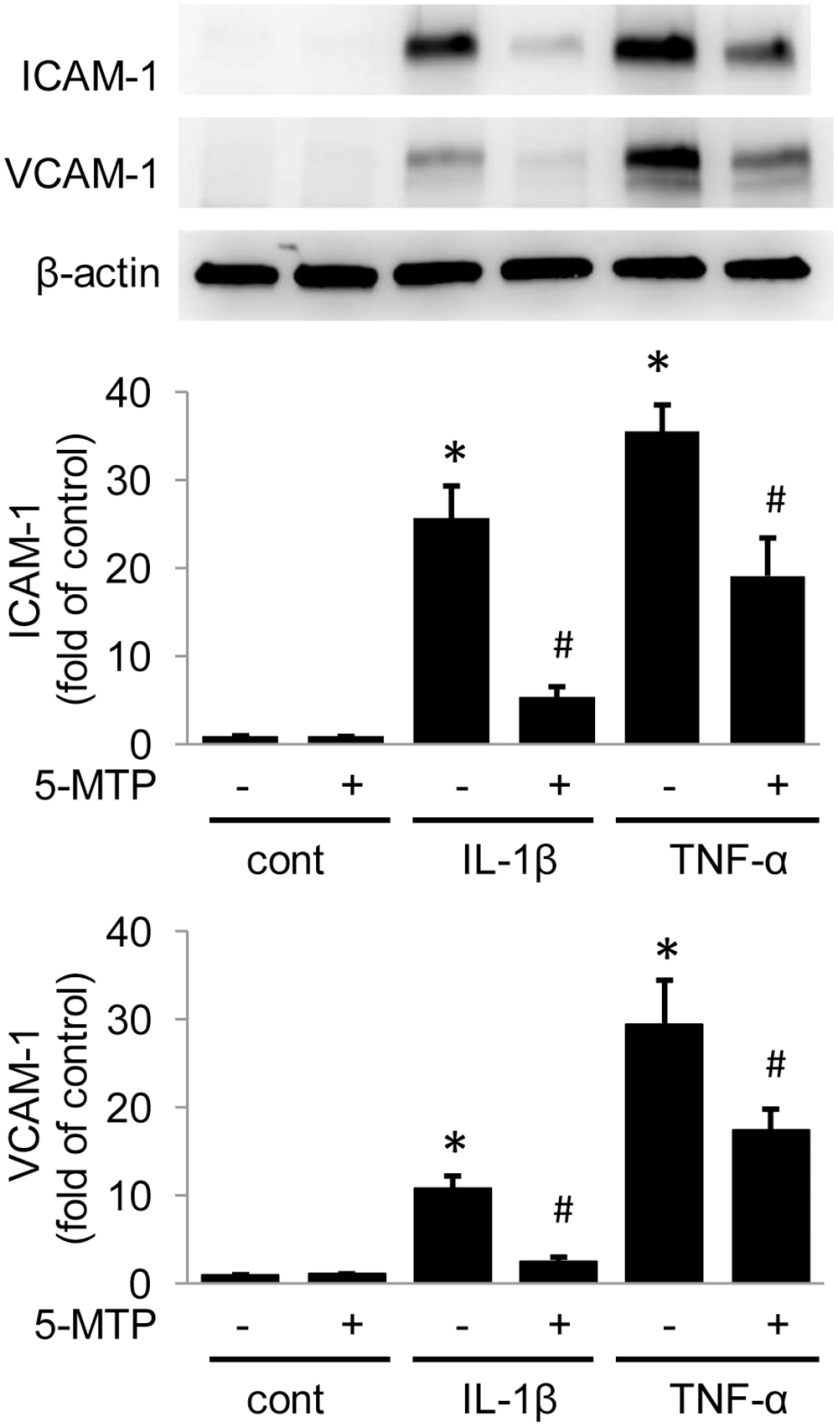
5-MTP suppresses pro-inflammatory cytokines-induced ICAM-1 and VCAM-1 expression. Analysis of ICAM-1 and VCAM-1 expression in HUVECs treated with IL-1β or TNF-α for 24 hours. Pretreatment with 5-MTP (100 μmol/L) attenuated pro-inflammatory cytokines-induced ICAM-1 and VCAM-1 expression. The upper panel shows representative blots and the lower panels, densitometry. Error bars denote mean ± SD (n = 3). * indicates *P* < .05 compared to control and # indicates *P* < .05 compared to cytokine alone group.

As cytokine-induced endothelial hyper-permeability contributes to expression of endothelial inflammatory molecules such as ICAM-1 and VCAM-1 which facilitate leukocyte adhesion and transmigration [22], we determined whether 5-MTP exerts an effect on the expression of these adhesion molecules. IL-1β- and TNFα- induced ICAM-1 or VCAM-1 expression was blunted by pretreatment with 5-MTP (Fig 7). These results suggest that 5-MTP controls not only the endothelial barrier function but also its inflammatory property.

### Pro-inflammatory Cytokines Suppress 5-MTP Production in HUVECs

**Fig 8:**
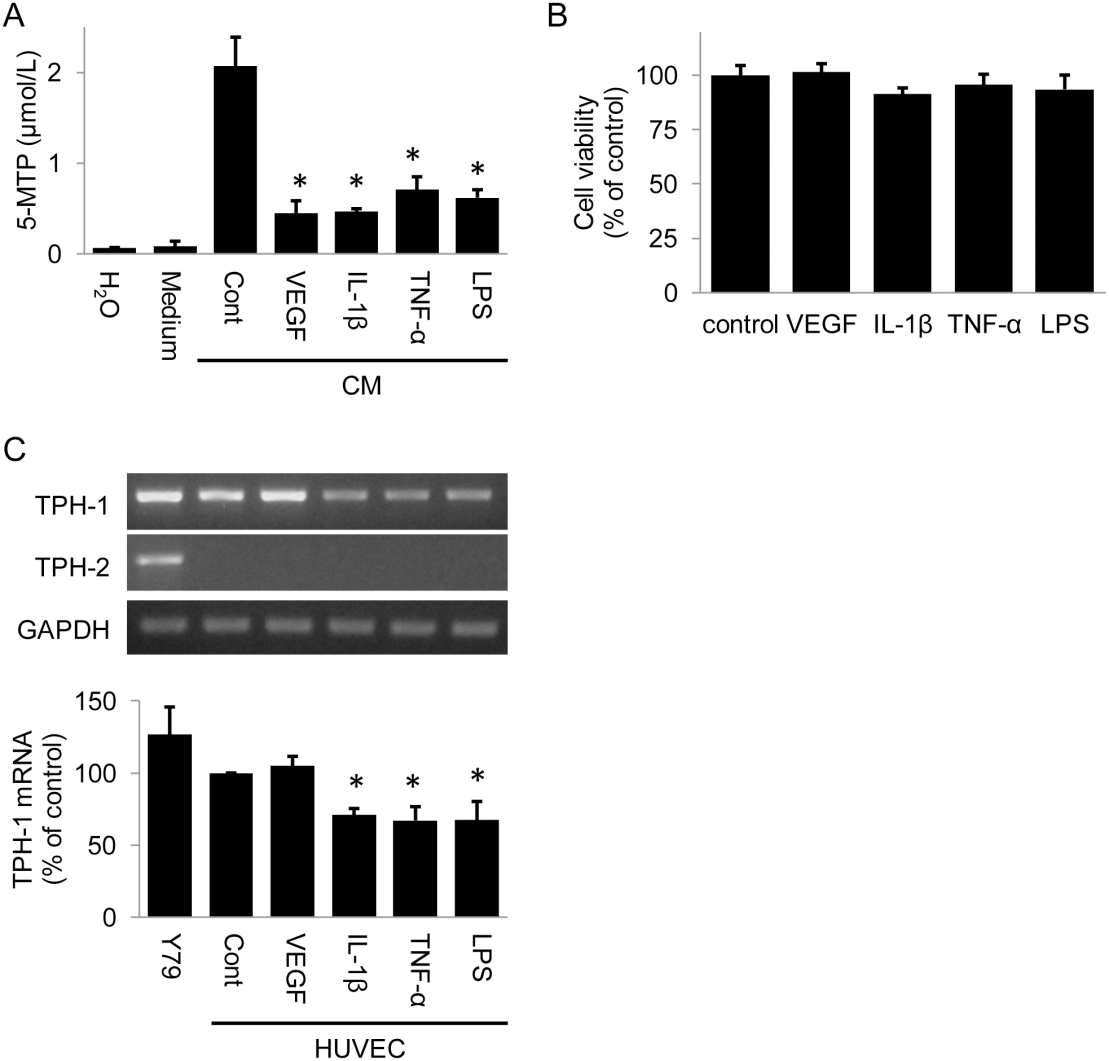
Pro-inflammatory cytokines downregulate 5-MTP production. (A) Measurement of 5-MTP in HUVEC-CM by ELISA. HUVECs were treated with VEGF or pro-inflammatory cytokines for 24 hours. H_2_O denotes deionized water, Medium, control medium and cont, vehicle control. * indicates *P* < .05 compared to control. (B) Cell vitality is not affected by vascular permeability factors. Cell survival was analyzed by MTT assay. (C) Analysis of TPH-1 and TPH-2 transcripts in HUVECs treated with VEGF or pro-inflammatory cytokines for 24 hours. TPH-1 and TPH-2 transcripts were measured by RT-PCR. Upper panel shows a representative gel and the lower panel, densitometry of three experiments. Y79 retinoblast cells were included as reference. Cont indicates vehicle control. Error bars denote mean ± SD of three experiments. * indicates *P* < .05 compared to control.

Since addition of 5-MTP rescued endothelial barrier function damaged by pro-inflammatory mediators, we wondered whether 5-MTP production is influenced by them. To determine this, we measured 5-MTP in the CM of HUVECs treated with VEGF, IL-1β, TNF-α or LPS. 5-MTP concentration in HUVEC-CM was reduced by each of the factors (Fig 8A). Reduction of 5-MTP is not due to cell death as neither VEGF, LPS, IL-1β nor TNF-α influenced HUVEC viability (Fig 8B). Tryptophan hydroxylase (TPH) is a pivotal enzyme in 5-MTP production. To determine whether downregulation of 5-MTP production by proinflammatory mediators is due to suppression of TPH, we analyzed TPH-1 and TPH-2 expressions in HUVECs by RT-PCR. HUVECs expressed TPH-1 but not TPH-2, while positive control retinoblastoma Y79 cells expressed both isoforms (Fig 8C). Interestingly, pro-inflammatory factors suppressed TPH-1 expression while VEGF had no effect (Fig 8C). These results suggest that pro-inflammatory factors downregulate 5-MTP production by suppressing TPH-1 expression.

## Discussion

The vascular endothelium defends against environmental insults by producing vasoprotective molecules, such as nitric oxide and prostacyclin that act in concert to maintain vascular tone, prevent platelet thrombosis and protect against endothelial apoptosis induced by pro-inflammatory mediators [23,24]. In this study, we demonstrated that a tryptophan metabolite, 5-MTP, is a novel endothelium-derived vasoprotective molecule. 5-MTP prevents endothelial hyperpermeability induced by VEGF, LPS, IL-1β and TNF-α through protection of VE-cadherin integrity via inhibition of p38 MAPK activation.

5-MTP was previously reported to be released by proliferative human fibroblasts but not quiescent fibroblasts or tumor cells [13,14]. Here we show that endothelial cell is another cell type that produces 5-MTP. Synthesis of 5-MTP requires TPH-catalyzed hydroxylation of L-tryptophan, which is well known as the rate-limiting step in biosynthesis of serotonin and melatonin [14,25]. Two isoforms of TPH, TPH-1 and TPH-2, have been identified [26]. We show in this study that endothelial cells express TPH-1 but not TPH-2, consistent with a previous report that TPH-1 is expressed in peripheral arteries while TPH-2 is selectively expressed in neural and pineal cells [27,28]. There is limited information about the regulation of TPH protein level probably because the TPH protein is unstable [29]. It was reported that TPH-1 mRNA is downregulated by hypoxia [30]. Our reports provide novel information about TPH-1 downregulation by pro-inflammatory mediators in endothelial cells which is accompanied by a large reduction of 5-MTP levels. Suppression of 5-MTP production via downregulation of TPH-1 expression by pro-inflammatory mediators accounts for endothelial hyperpermeability that occurs during inflammatory challenges. These findings suggest that TPH-1/5-MTP pathway plays a critical role in defending against vascular dysfunction and inflammation.

Adherens junction composed by homotypic adhesion of VE-cadherin plays prominent role in endothelial barrier function [31]. Disruption of adherens junction leads to endothelial hyperpermeability [32,33]. Surface expression of VE-cadherin has been shown to be downregulated by various pro-inflammatory mediators such as VEGF, TNF-α and LPS [5,8,9]. Expression of total VE-cadherin in HUVECs has also been shown to be downregulated by IL-1β via a Rac-dependent mechanism [34]. Here we show p38 MAPK is required for various pro-inflammatory factors to downregulate the expression of VE-cadherin. p38 MAPK is recognized as a key signaling pathway via which diverse pro-inflammatory mediators, environmental toxins, and oxidants disrupt the barrier function and increase vascular permeability [18–20,35]. We show that 5-MTP does not affect basal phosphorylation levels of p38 MAPK; however, in response to inflammatory mediator stimulations, 5-MTP significantly reduces p38 MAPK phosphorylation. It is unclear how 5-MTP blocks p38 MAPK activation. It has been reported that p38 MAPK is endogenously controlled by phosphatases including MAPK phosphatase-1 (MKP-1) and protein phosphatase-2C, WIP-1 [36,37]. Induction of MKP-1 expression was reported to block TNF-α induced vascular hyperpermeability through inhibition of p38 MAPK activation [36,38]. The possibility that 5-MTP protects endothelial barrier function by inducing MKP-1 expression remains to be determined. Although Src family kinases are intimately involved in signaling VE-cadherin dysfunction and increased vascular permeability induced by VEGF [39], recent reports show that Src-induced VE-cadherin phosphorylation is not sufficient for disrupting endothelial barrier function [40]. Our finding of p38 MAPK as a key pathway employed not only by cytokines and LPS but also by VEGF to downregulate VE-cadherin expression is in keeping with this notion. It is unclear how p38 MAPK activation induced by diverse pro-inflammatory mediators leads to reduced expression of VE-cadherin. VE-cadherin expression is regulated at the transcriptional level by Wnt [41] and post-translationally by internalization and degradation, which depends on Src signaling pathway [5]. p38 MAPK activation has not been linked to suppression of VE-cadherin transcription or internalization. However, it was reported that p38 MAPK regulates opioid receptor endocytosis [42]. Further studies will be needed to elucidate the mechanism by which p38 MAPK activation alters VE-cadherin expression.

5-MTP and melatonin share common structural features and biological activities. Structurally, 5-MTP and melatonin share a common 5-methoxy moiety at the indole backbone and functionally, they inhibit COX-2 expression induced by pro-inflammatory mediators through blocking p300 HAT and NF-κB activation although a higher concentration is required for melatonin to exert this action [16]. A recent report suggests that melatonin inhibits IL-1β induced endothelial hyperpermeability [33]. These findings suggest that 5-MTP and melatonin may act via similar receptor and signaling pathways. We have entertained the possibility that 5-MTP acts via interactions with plasma membrane receptors. Work is in progress to ascertain the expression of 5-MTP receptors on cell surface and determine whether melatonin competes with 5-MTP on receptor binding.

In summary, we have identified 5-MTP as a novel metabolite of L-tryptophan produced by endothelial cells. 5-MTP plays an important role in protecting VE-cadherin and thereby defending against endothelial barrier disruption by pro-inflammatory mediators through inhibiting p38 MAPK activation. 5-MTP is considered as a new class of endothelium-derived molecules which protects endothelial barrier function and defends against vascular leakage and inflammation.

## References

[1] Dejana E. Endothelial cell-cell junctions: Happy together. Nat Rev Mol Cell Biol. 2004;5:261–270. 15071551 10.1038/nrm1357

[2] Dejana E, Orsenigo F, Lampugnani MG. The role of adherens junctions and VE-cadherin in the control of vascular permeability. J Cell Sci. 2008;121:2115–2122. 10.1242/jcs.017897 18565824

[3] Vestweber D. VE-cadherin: the major endothelial adhesion molecule controlling cellular junctions and blood vessel formation. Arterioscler Thromb Vasc Biol. 2008;28:223–232. 18162609 10.1161/ATVBAHA.107.158014

[4] Senger DR, Galli SJ, Dvorak AM, Perruzzi CA, Harvey VS, Dvorak HF. Tumor cells secrete a vascular permeability factor that promotes accumulation of ascites fluid. Science. 1983;219:983–985. 6823562 10.1126/science.6823562

[5] Gavard J, Gutkind JS. VEGF controls endothelial-cell permeability by promoting the β-arrestin-dependent endocytosis of VE-cadherin. Nat Cell Biol. 2006;8:1223–1234. 17060906 10.1038/ncb1486

[6] Eliceiri BP, Paul R, Schwartzberg PL, Hood JD, Leng J, Cheresh DA. Selective requirement for Src kinases during VEGF-induced angiogenesis and vascular permeability. Mol cell. 1999;4:915–924. 10635317 10.1016/s1097-2765(00)80221-x

[7] Weis S, Cui J, Barnes L, Cheresh D. Endothelial barrier disruption by VEGF-mediated Src activity potentiates tumor cell extravasation and metastasis. J Cell Biol. 2004;167:223–229. 15504909 10.1083/jcb.200408130 PMC2172541

[8] Xing J, Birukova AA. ANP attenuates inflammatory signaling and Rho pathway of lung endothelial permeability induced by LPS and TNFα. Microvasc Res. 2010;79:56–62. 10.1016/j.mvr.2009.11.006 19931545 PMC2813389

[9] Nwariaku FE, Chang J, Zhu X, Liu Z, Duffy SL, Halaihel NH, et al The role of p38 map kinase in tumor necrosis factor-induced redistribution of vascular endothelial cadherin and increased endothelial permeability. Shock. 2002;18:82–85. 12095140 10.1097/00024382-200207000-00015

[10] Libby P, Ridker PM, Maseri A. Inflammation and atherosclerosis. Circulation. 2002;105:1135–1143. 11877368 10.1161/hc0902.104353

[11] Hansson GK, Hermansson A. The immune system in atherosclerosis. Nat Immunol. 2011;12:204–212. 10.1038/ni.2001 21321594

[12] Deng WG, Saunders M, Gilroy D, He XZ, Yeh H, Zhu Y, et al Purification and characterization of a cyclooxygenase-2 and angiogenesis suppressing factor produced by human fibroblasts. FASEB J. 2002;16:1286–1288. 12060668 10.1096/fj.01-0844fje

[13] Cheng HH, Wang KH, Chu LY, Chang TC, Kuo CC, Wu KK. Quiescent and proliferative fibroblasts exhibit differential p300 hat activation through control of 5-methoxytryptophan production. PLoS One. 2014;9:e88507 10.1371/journal.pone.0088507 24523905 PMC3921189

[14] Cheng HH, Kuo CC, Yan JL, Chen HL, Lin WC, Wang KH, et al Control of cyclooxygenase-2 expression and tumorigenesis by endogenous 5-methoxytryptophan. Proc Natl Acad Sci U S A. 2012;109:13231–13236. 10.1073/pnas.1209919109 22851770 PMC3421199

[15] Wu KK, Cheng HH, Chang TC. 5-methoxyindole metabolites of l-tryptophan: Control of cox-2 expression, inflammation and tumorigenesis. J Biomed Sci. 2014;21:17 10.1186/1423-0127-21-17 24589238 PMC3975872

[16] Deng WG, Tang ST, Tseng HP, Wu KK. Melatonin suppresses macrophage cyclooxygenase-2 and inducible nitric oxide synthase expression by inhibiting p52 acetylation and binding. Blood. 2006;108:518–524. 16609073 10.1182/blood-2005-09-3691 PMC1895491

[17] Niwa K, Inanami O, Ohta T, Ito S, Karino T, Kuwabara M. P38 mapk and Ca^2+^ contribute to hydrogen peroxide-induced increase of permeability in vascular endothelial cells but ERK does not. Free Radic Res. 2001;35:519–527. 11767410 10.1080/10715760100301531

[18] Borbiev T, Birukova A, Liu F, Nurmukhambetova S, Gerthoffer WT, Garcia JG, et al P38 MAP kinase-dependent regulation of endothelial cell permeability. Am J Physiol Lung Cell Mol Physiol. 2004;287:L911–918. 15475493 10.1152/ajplung.00372.2003

[19] Liu T, Milia E, Warburton RR, Hill NS, Gaestel M, Kayyali US. Anthrax lethal toxin disrupts the endothelial permeability barrier through blocking p38 signaling. J Cell Physiol. 2012;227:1438–1445. 10.1002/jcp.22859 21618534 PMC4254851

[20] Dong F, Guo F, Li L, Guo L, Hou Y, Hao E, et al Cadmium induces vascular permeability via activation of the p38 MAPK pathway. Biochem Biophys Res Commun. 2014;450:447–452. 10.1016/j.bbrc.2014.05.140 24909688

[21] Koss M, Pfeiffer GR 2nd, Wang Y, Thomas ST, Yerukhimovich M, Gaarde WA, et al Ezrin/radixin/moesin proteins are phosphorylated by TNF-α and modulate permeability increases in human pulmonary microvascular endothelial cells. J Immunol. 2006;176:1218–1227. 16394012 10.4049/jimmunol.176.2.1218

[22] Min JK, Kim YM, Kim SW, Kwon MC, Kong YY, Hwang IK, et al TNF-related activation-induced cytokine enhances leukocyte adhesiveness: induction of ICAM-1 and VCAM-1 via TNF receptor-associated factor and protein kinase C-dependent NF-kappaB activation in endothelial cells. J Immunol. 2005; 175, 531–540. 15972689 10.4049/jimmunol.175.1.531

[23] Liou JY, Lee S, Ghelani D, Matijevic-Aleksic N, Wu KK. Protection of endothelial survival by peroxisome proliferator-activated receptor-δ mediated 14-3-3 upregulation. Arterioscler Thromb Vasc Biol. 2006;26:1481–1487. 16645156 10.1161/01.ATV.0000223875.14120.93

[24] Chu LY, Liou JY, Wu KK. Prostacyclin protects vascular integrity via PPAR/14-3-3 pathway. Prostaglandins & other lipid mediator. 2015;118–119c:19–27. 10.1016/j.prostaglandins.2015.04.006 25910681

[25] Lovenberg W, Jequier E, and Sjoerdsma A. Tryptophan hydroxylation: measurement in pineal gland, brainstem, and carcinoid tumor. Science. 1967;155: 217–19. 6015530 10.1126/science.155.3759.217

[26] Darmon MC, Guibert B, Leviel V, Ehret M, Maitre M, and Mallet J. Sequence of two mRNAs encoding activity of rat tryptophan hydroxylase. J. Neurochem. 1988;51: 312–16. 3379411 10.1111/j.1471-4159.1988.tb04871.x

[27] Ni W, Geddes TJ, Priestley JR, Szasz T, Kuhn DM, Watts SW. The existence of a local 5-hydroxytryptaminergic system in peripheral arteries. Br J Pharmacol. 2008;154:663–674. 10.1038/bjp.2008.111 18414394 PMC2439511

[28] Walther DJ, Peter JU, Bashammakh S, Hortnagl H, Voits M, Fink H, et al Synthesis of serotonin by a second tryptophan hydroxylase isoform. Science. 2003;299: 76 12511643 10.1126/science.1078197

[29] Cash CD. Why tryptophan hydroxylase is difficult to purify: a reactive oxygen derived species-mediated phenomenon that may be implicated in human pathology. Gen. Pharmacol. 1998;30: 569–74. 9522177 10.1016/s0306-3623(97)00308-x

[30] Rahman MS, and Thomas P. Molecular cloning, characterization and expression of two tryptophan hydroxylase (TPH-1 and TPH-2) genes in the hypothalamus of Atlantic croaker: Down-regulation after chronic exposure to hypoxia. Neuroscience. 2009;158: 751–65. 10.1016/j.neuroscience.2008.10.029 19015006

[31] Dejana E, Giampietro C. Vascular endothelial-cadherin and vascular stability. Curr Opin Hematol. 2012;19:218–223. 10.1097/MOH.0b013e3283523e1c 22395663

[32] Giannotta M, Trani M, Dejana E. VE-cadherin and endothelial adherens junctions: active guardians of vascular integrity. Dev Cell. 2013;26:441–454. 10.1016/j.devcel.2013.08.020 24044891

[33] Broman MT, Kouklis P, Gao X, Ramchandran R, Neamu RF. Minshall RD, et al Cdc42 regulates adherens junction stability and endothelial permeability by inducing alpha-catenin interaction with the vascular endothelial cadherin complex. Circ Res. 2006;98:73–80. 16322481 10.1161/01.RES.0000198387.44395.e9

[34] Yuan X, Li B, Li H, Xiu R. Melatonin inhibits IL-1β-induced monolayer permeability of human umbilical vein endothelial cells via Rac activation. J Pineal Res. 2011;51:220–5. 10.1111/j.1600-079X.2011.00882.x 21535449

[35] Goldberg PL, MacNaughton DE, Clements RT, Minnear FL, Vincent PA. P38 MAPK activation by TGF-β1 increases MLC phosphorylation and endothelial monolayer permeability. Am J Physiol Lung Cell Mol Physiol. 2002;282:L146–154. 11741826 10.1152/ajplung.2002.282.1.L146

[36] Kiemer AK, Weber NC, Furst R, Bildner N, Kulhanek-Heinze S, Vollmar AM. Inhibition of p38 mapk activation via induction of mkp-1: Atrial natriuretic peptide reduces TNF-α-induced actin polymerization and endothelial permeability. Circ Res. 2002;90:874–881. 11988488 10.1161/01.res.0000017068.58856.f3

[37] Takekawa M, Adachi M, Nakahata A, Nakayama I, Itoh F, Tsukuda H, et al p53-inducible Wip1 phosphatase mediates a negative feedback regulation of p38 MAPK-p53 signaling in response to UV radiation. EMBO J. 2000;19:6517–6526. 11101524 10.1093/emboj/19.23.6517 PMC305857

[38] Yang D, Xie P, Guo S, Li H. Induction of MAPK phosphatase-1 by hypothermia inhibits TNF-α-induced endothelial barrier dysfunction and apoptosis. Cardiovasc Res. 2010;85:520–529. 10.1093/cvr/cvp323 19793766

[39] Gong P, Angelini DJ, Yang S, Xia G, Cross AS, Mann D, et al Tlr4 signaling is coupled to src family kinase activation, tyrosine phosphorylation of zonula adherens proteins, and opening of the paracellular pathway in human lung microvascular endothelia. J Biol Chem. 2008;283:13437–13449. 10.1074/jbc.M707986200 18326860 PMC2442341

[40] Adam AP, Sharenko AL, Pumiglia K, Vincent PA. Src-induced tyrosine phosphorylation of VE-cadherin is not sufficient to decrease barrier function of endothelial monolayers. J Biol Chem. 2010;285:7045–7055. 10.1074/jbc.M109.079277 20048167 PMC2844154

[41] Cowan CE, Kohler EE, Dugan TA, Mirza MK, Malik AB, Wary KK. Kruppel-like factor-4 transcriptionally regulates VE-cadherin expression and endothelial barrier function. Circ Res. 2010;107:959–966. 10.1161/CIRCRESAHA.110.219592 20724706 PMC3018700

[42] Mace G, Miaczynska M, Zerial M, Nebreda AR. Phosphorylation of EEA1 by p38 MAP kinase regulates mu opioid receptor endocytosis. EMBO J. 2005;24:3235–3246. 16138080 10.1038/sj.emboj.7600799 PMC1224689

